# Dimethyl 1-(4-methyl­phen­yl)-8-(thio­phen-2-yl)-11-oxatricyclo­[6.2.1.0^2,7^]undeca-2,4,6,9-tetra­ene-9,10-dicarboxy­late

**DOI:** 10.1107/S1600536813005308

**Published:** 2013-03-06

**Authors:** B. Balakrishnan, Meganathan Nandakumar, Pandamangalam R. Seshadri, Arasambattu K. Mohanakrishnan

**Affiliations:** a Department of Physics, P.T. Lee Chengalvaraya Naicker College of Engineering & Technology, Kancheepuram 631 502, India; b Department of Organic Chemistry, University of Madras, Guindy Campus, Chennai 600 025, India; c PostGraduate & Research Department of Physics, Agurchand Manmull Jain College, Chennai 600 114, India

## Abstract

The title compound, C_25_H_20_O_5_S, is the product of a Diels–Alder reaction. The mol­ecule consists of a fused tricyclic system containing two five-membered rings and one six-membered ring. The five-membered rings both show an envelope conformation with the O atom at the flap, whereas the six-membered ring adopts a boat conformation. The thio­phene ring is disordered over two sets of sites with an occupancy ratio of 0.53 (1):0.47 (1). The dihedral angles between the 4-methyl­phenyl ring and the major and minor components of the thio­phene ring are 66.3 (1) and 67.9 (1)°, respectively, while the dihedral angle between the disordered thio­phenyl components is 3.1 (1)°. The mean plane of the tricyclic ring system makes dihedral angles of 35.8 (1), 30.8 (1) and 32.8 (1)°, respectively, with the 4-methyl­phenyl ring and the major and minor components of the thio­phenyl ring. In the crystal, inversion dimers are formed through pairs of C—H⋯π inter­actions. In addition, C—H⋯O inter­actions are observed.

## Related literature

For background to Diels–Alder reactions, see: Denmark & Thorarensen (1996 ▶). For related structures, see: Ohwada *et al.* (2001 ▶); Takahashi *et al.* (2003 ▶); Fun *et al.* (2011 ▶); Gurbanov *et al.* (2009 ▶); Balakrishnan *et al.* (2013 ▶). For puckering and asymmetry parameters, see: Cremer & Pople (1975 ▶); Nardelli (1983 ▶).
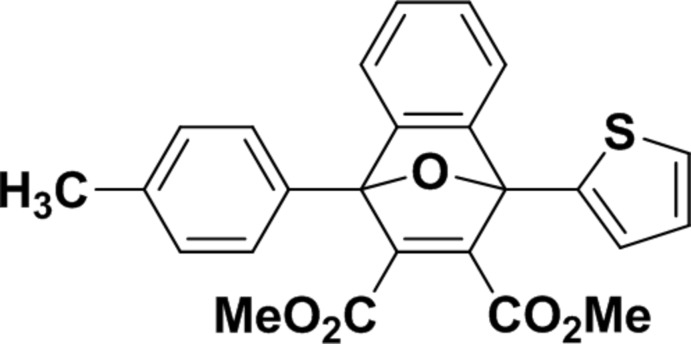


## Experimental

### 

#### Crystal data

C_25_H_20_O_5_S
*M* *_r_* = 432.47
Triclinic, 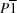

*a* = 7.5966 (15) Å
*b* = 10.877 (2) Å
*c* = 13.515 (3) Å
α = 91.339 (5)°
β = 93.456 (4)°
γ = 100.129 (5)°
*V* = 1096.6 (4) Å^3^
*Z* = 2
Mo *K*α radiation
μ = 0.18 mm^−1^
*T* = 293 K
0.35 × 0.30 × 0.25 mm

#### Data collection

Bruker Kappa APEXII CCD diffractometer
19981 measured reflections
5464 independent reflections
4142 reflections with *I* > 2σ(*I*)
*R*_int_ = 0.024

#### Refinement

*R*[*F* ^2^ > 2σ(*F* ^2^)] = 0.041
*wR*(*F* ^2^) = 0.129
*S* = 1.03
5464 reflections
321 parameters
52 restraints
H-atom parameters constrained
Δρ_max_ = 0.25 e Å^−3^
Δρ_min_ = −0.16 e Å^−3^

### 

Data collection: *APEX2* (Bruker, 2008 ▶); cell refinement: *SAINT* (Bruker, 2008 ▶); data reduction: *SAINT*; program(s) used to solve structure: *SHELXS97* (Sheldrick, 2008 ▶); program(s) used to refine structure: *SHELXL97* (Sheldrick, 2008 ▶); molecular graphics: *ORTEP-3 for Windows* (Farrugia, 2012 ▶); software used to prepare material for publication: *SHELXL97*, *PLATON* (Spek, 2009 ▶) and *publCIF* (Westrip, 2010 ▶).

## Supplementary Material

Crystal structure: contains datablock(s) I, global. DOI: 10.1107/S1600536813005308/im2420sup1.cif

Structure factors: contains datablock(s) I. DOI: 10.1107/S1600536813005308/im2420Isup2.hkl

Supplementary material file. DOI: 10.1107/S1600536813005308/im2420Isup3.cml

Additional supplementary materials:  crystallographic information; 3D view; checkCIF report

## Figures and Tables

**Table 1:** Hydrogen-bond geometry (Å, °) *Cg* is the centroid of the C2–C7 ring.

*D*—H⋯*A*	*D*—H	H⋯*A*	*D*⋯*A*	*D*—H⋯*A*
C11—H11⋯O4^i^	0.93	2.47	3.378 (1)	165
C17—H17*B*⋯*Cg* ^ii^	0.96	3.26	3.99 (2)	136

Symmetry codes: (i) 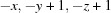; (ii) 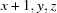.

**Fig. 1.**
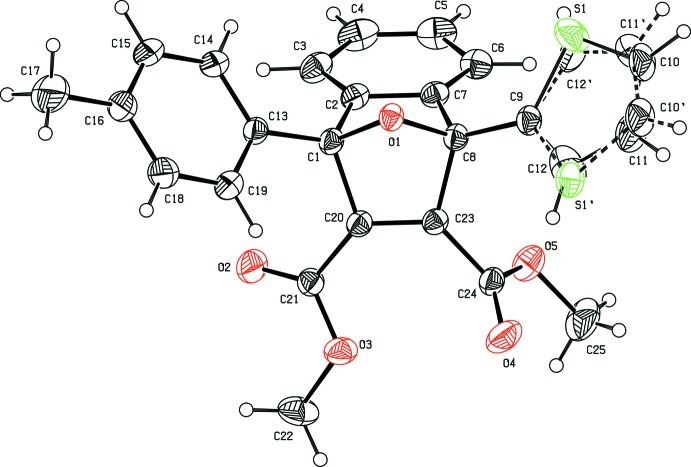
Molecular structure of the title compound, showing 30% probability displacement ellipsoids.

**Fig. 2.**
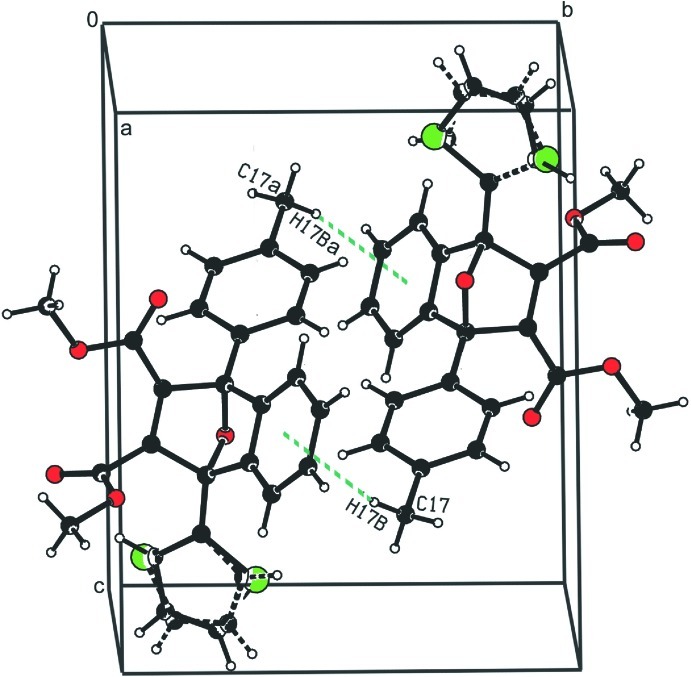
A view of the C—H···π interactions in the crystal structure of the title compound.

## supplementary crystallographic information

### Comment

The Diels-Alder reaction involves [4 + 2] cycloaddition of a conjucated diene and a dienophile (an alkene or alkyne). The Diels-Alder reaction is among the most powerful C—C bond forming process and one of most widely used and studied transformation in organic chemistry (Denmark & Thorarensen, 1996). The title compound, C_25_H_20_O_5_S, comprises a fused tricyclic system with one 4-methylphenyl and one thiophenyl group attached to it. The tricyclic system consists of two 5-membered rings and one aromatic ring. In addition, two carboxylate units are attached to the tricyclic system. Geometrical parameters agree well with reported structures (Fun *et al.*, 2011; Gurbanov *et al.*, 2009; Ohwada *et al.* 2001; Takahashi *et al.* 2003). The five membered ring C_1_\C_2_\C_7_\C_8_\O_1_ adopts an envelope conformation with atom O_1_ displaced by 0.787 Å from the mean plane of the other ring atoms C_1_\C_2_\C_7_\C_8_.The puckering parameters (Cremer & Pople, 1975) and asymmetry parameters (Nardelli, 1983) are q_2_ = 0.539 (1) Å, φ = 144.8 (1)°, Δ_S_(O_1_) = 0.006 (1)° and Δ_2_(O_1_) = 0.325 (1)°.The second five membered ring C_1_\ C_20_\C_23_\C_8_\O_1_ also adopts an envelope conformation with O_1_ displaced by -0.787 Å from the mean plane of the other ring atoms C_1_\ C_20_\C_23_\C_8_.The puckering parameters (Cremer & Pople, 1975) and asymmetry parameters (Nardelli, 1983) are q_2_ = 0.542 (1) Å, φ = -37.3 (1)°, Δ_S_(O_1_) = 0.011 (1)° and Δ_2_(O_1_) = 0.327 (1)°.The six membered ring C_1_/C_2_/C_7_/C_8_/C_23_/C_20_ adopts boat conformation with puckering parameter q_2_ = 0.9849 (1) Å, θ= 89.9 (8)° and φ = 359.2 (8)°.

The thiophene ring is disordered over two sites with occupancy ratio of 0.53 (3): 0.47 (3).The dihedral angle between the rings C_1_/C_2_/C_7_/C_8_/O_1_ and C_1_/C_20_/C_23_/C_8_/O_1_ is 82.15 (1)°. The dihedral angle between the terminal 4-methylphenyl and major and minor components of the thiophene rings are 66.3 (1)° and 67.9 (1)° respectively. The mean plane of the tricyclic system makes dihedral angles of 35.8 (1)°, 30.8 (1)° and 32.8 (1)°, respectively, with the 4-methylphenyl ring and the major and minor components of the thiophenyl group. The carboxylate ligand at the C20 carbon atom is turned out the plane in the positive direction of the five membered ring C20/ C1/ O1/ C8/ C23 (the torsion angle C23—C20—C21—O3= 32.2 (2)°, while that at the C23 carbon atom is turned out of this plane in the negative direction (the torsion angle is C20—C23—C24=O4 = -73.8 (2)°). In the crystal structure centrosymmetric dimers are realised by C—H···π interactions. In addition, intermolecular C—H···O interactions are observed (Table 1).

### Experimental

To a stirred solution of 1-(thiophen-2-yl)-3-*p*-tolylisobenzofuran (2 g, 6.897 mmol) in dry DCM was added DMAD (1.08 g, 7.59 mmol) and the reaction mixture was stirred for 0.5 h at room temperature under nitrogen atmosphere. The solvent was removed and the resulting solid was washed with methanol to give the title compound as a colourless solid. This adduct was crystallized from CHCl_3_/CH_3_OH (3:1) by slow evaporation method.

### Refinement

All H atoms were positioned geometrically and allowed to ride on their parent atoms, with (C—H= 0.93–0.96 Å), and *U*_iso_(H) = 1.5 *U*_eq_(C) for methyl H atoms and 1.2 Ueq(C) for other H atoms.The thiophene ring is disordered over two sites with occupancy ratio of 0.53 (3): 0.47 (3).

### Crystal data

**Table:**

C_25_H_20_O_5_S	*V* = 1096.6 (4) Å^3^
*M**_r_* = 432.47	*Z* = 2
Triclinic, *P*1	*F*(000) = 452
Hall symbol: -P 1	*D*_x_ = 1.310 Mg m^−^^3^
*a* = 7.5966 (15) Å	Mo *K*α radiation, λ = 0.71073 Å
*b* = 10.877 (2) Å	θ = 1.5–28.5°
*c* = 13.515 (3) Å	µ = 0.18 mm^−^^1^
α = 91.339 (5)°	*T* = 293 K
β = 93.456 (4)°	Block, colourless
γ = 100.129 (5)°	0.35 × 0.30 × 0.25 mm

### Data collection

**Table:**

Bruker Kappa APEXII CCD diffractometer	4142 reflections with *I* > 2σ(*I*)
Radiation source: fine-focus sealed tube	*R*_int_ = 0.024
Graphite monochromator	θ_max_ = 28.5°, θ_min_ = 1.5°
ω and φ scans	*h* = −10→10
19981 measured reflections	*k* = −14→14
5464 independent reflections	*l* = −18→18

### Refinement

**Table:**

Refinement on *F*^2^	Primary atom site location: structure-invariant direct methods
Least-squares matrix: full	Secondary atom site location: difference Fourier map
*R*[*F*^2^ > 2σ(*F*^2^)] = 0.041	Hydrogen site location: inferred from neighbouring sites
*wR*(*F*^2^) = 0.129	H-atom parameters constrained
*S* = 1.03	*w* = 1/[σ^2^(*F*_o_^2^) + (0.0648*P*)^2^ + 0.1968*P*] where *P* = (*F*_o_^2^ + 2*F*_c_^2^)/3
5464 reflections	(Δ/σ)_max_ < 0.001
321 parameters	Δρ_max_ = 0.25 e Å^−^^3^
52 restraints	Δρ_min_ = −0.16 e Å^−^^3^

### Special details

**Table:**

Geometry. All e.s.d.'s (except the e.s.d. in the dihedral angle between two l.s. planes) are estimated using the full covariance matrix. The cell e.s.d.'s are taken into account individually in the estimation of e.s.d.'s in distances, angles and torsion angles; correlations between e.s.d.'s in cell parameters are only used when they are defined by crystal symmetry. An approximate (isotropic) treatment of cell e.s.d.'s is used for estimating e.s.d.'s involving l.s. planes.
Refinement. Refinement of *F*^2^ against ALL reflections. The weighted *R*-factor *wR* and goodness of fit *S* are based on *F*^2^, conventional *R*-factors *R* are based on *F*, with *F* set to zero for negative *F*^2^. The threshold expression of *F*^2^ > σ(*F*^2^) is used only for calculating *R*-factors(gt) *etc*. and is not relevant to the choice of reflections for refinement. *R*-factors based on *F*^2^ are statistically about twice as large as those based on *F*, and *R*- factors based on ALL data will be even larger.

### Fractional atomic coordinates and isotropic or equivalent isotropic displacement parameters (Å^2^)

**Table:**

	*x*	*y*	*z*	*U*_iso_*/*U*_eq_	Occ. (<1)
C17	−0.3508 (4)	−0.3528 (2)	0.05041 (17)	0.1061 (10)	
H17A	−0.2958	−0.4245	0.0396	0.159*	
H17B	−0.3950	−0.3260	−0.0121	0.159*	
H17C	−0.4485	−0.3742	0.0923	0.159*	
C16	−0.2140 (3)	−0.24828 (15)	0.09987 (12)	0.0618 (5)	
C18	−0.2271 (2)	−0.21039 (15)	0.19687 (12)	0.0577 (4)	
H18	−0.3214	−0.2500	0.2319	0.069*	
C19	−0.1036 (2)	−0.11537 (14)	0.24304 (11)	0.0484 (3)	
H19	−0.1157	−0.0918	0.3085	0.058*	
C13	0.03822 (19)	−0.05453 (11)	0.19297 (9)	0.0391 (3)	
C14	0.0529 (2)	−0.09272 (14)	0.09555 (10)	0.0520 (4)	
H14	0.1476	−0.0537	0.0605	0.062*	
C15	−0.0721 (3)	−0.18817 (16)	0.05039 (11)	0.0647 (5)	
H15	−0.0601	−0.2125	−0.0149	0.078*	
C1	0.15925 (18)	0.05759 (11)	0.24036 (9)	0.0365 (3)	
C2	0.34514 (18)	0.10769 (12)	0.20407 (9)	0.0394 (3)	
C3	0.4745 (2)	0.05272 (16)	0.16187 (11)	0.0510 (4)	
H3	0.4534	−0.0321	0.1446	0.061*	
C4	0.6378 (2)	0.12840 (19)	0.14586 (12)	0.0622 (5)	
H4	0.7257	0.0938	0.1160	0.075*	
C5	0.6713 (2)	0.25310 (19)	0.17340 (13)	0.0608 (4)	
H5	0.7816	0.3014	0.1622	0.073*	
C6	0.5419 (2)	0.30857 (15)	0.21812 (11)	0.0494 (3)	
H6	0.5653	0.3926	0.2381	0.059*	
C7	0.37929 (18)	0.23483 (12)	0.23151 (9)	0.0391 (3)	
C8	0.21057 (17)	0.25712 (11)	0.28215 (9)	0.0359 (3)	
C23	0.21701 (17)	0.18743 (11)	0.38086 (9)	0.0358 (3)	
C20	0.18841 (18)	0.06611 (12)	0.35510 (9)	0.0364 (3)	
C21	0.23243 (19)	−0.03877 (12)	0.41371 (10)	0.0409 (3)	
C22	0.2710 (3)	−0.11567 (19)	0.57314 (13)	0.0660 (5)	
H22A	0.2071	−0.1960	0.5492	0.099*	
H22B	0.2407	−0.1000	0.6397	0.099*	
H22C	0.3975	−0.1145	0.5725	0.099*	
C24	0.2738 (2)	0.25025 (12)	0.47907 (10)	0.0413 (3)	
C25	0.5151 (3)	0.3704 (2)	0.57359 (15)	0.0837 (7)	
H25A	0.4399	0.4267	0.5952	0.126*	
H25B	0.6332	0.4162	0.5656	0.126*	
H21C	0.5214	0.3081	0.6222	0.126*	
C9	0.16129 (19)	0.38335 (12)	0.28373 (10)	0.0409 (3)	
O2	0.2780 (2)	−0.12775 (11)	0.37594 (9)	0.0698 (4)	
O3	0.22201 (16)	−0.01996 (10)	0.50989 (7)	0.0536 (3)	
O4	0.17981 (19)	0.24950 (13)	0.54677 (9)	0.0712 (4)	
O5	0.44061 (16)	0.31032 (11)	0.47947 (8)	0.0606 (3)	
O1	0.07523 (12)	0.16735 (8)	0.22614 (6)	0.0360 (2)	
S1	0.2161 (5)	0.4865 (3)	0.1956 (3)	0.0697 (7)	0.531 (3)
C12	0.0495 (16)	0.4293 (11)	0.3505 (9)	0.093 (5)	0.531 (3)
H12	0.0023	0.3858	0.4041	0.112*	0.531 (3)
C11	0.019 (3)	0.5478 (14)	0.3260 (14)	0.066 (2)	0.531 (3)
H11	−0.0536	0.5916	0.3607	0.079*	0.531 (3)
C10	0.1054 (17)	0.5919 (11)	0.2473 (9)	0.063 (2)	0.531 (3)
H10	0.1048	0.6714	0.2235	0.076*	0.531 (3)
S1'	0.0250 (4)	0.4286 (3)	0.3645 (2)	0.0532 (4)	0.466 (3)
C12'	0.214 (2)	0.4812 (10)	0.2173 (8)	0.071 (4)	0.466 (3)
H12'	0.2990	0.4796	0.1708	0.085*	0.466 (3)
C11'	0.122 (2)	0.5793 (14)	0.2312 (12)	0.082 (3)	0.466 (3)
H11'	0.1272	0.6462	0.1892	0.098*	0.466 (3)
C10'	0.024 (4)	0.5679 (16)	0.3107 (17)	0.063 (3)	0.466 (3)
H10'	−0.0362	0.6289	0.3338	0.076*	0.466 (3)

### Atomic displacement parameters (Å^2^)

**Table:**

	*U*^11^	*U*^22^	*U*^33^	*U*^12^	*U*^13^	*U*^23^
C17	0.132 (2)	0.0921 (16)	0.0677 (12)	−0.0455 (15)	−0.0117 (13)	−0.0130 (11)
C16	0.0819 (12)	0.0487 (8)	0.0460 (8)	−0.0072 (8)	−0.0117 (8)	0.0001 (6)
C18	0.0628 (10)	0.0538 (9)	0.0509 (8)	−0.0046 (7)	0.0019 (7)	0.0017 (7)
C19	0.0553 (9)	0.0477 (8)	0.0410 (7)	0.0068 (6)	0.0034 (6)	−0.0043 (6)
C13	0.0477 (8)	0.0339 (6)	0.0363 (6)	0.0110 (5)	−0.0027 (5)	−0.0017 (5)
C14	0.0696 (10)	0.0478 (8)	0.0349 (7)	0.0001 (7)	0.0030 (6)	−0.0001 (6)
C15	0.0943 (14)	0.0575 (9)	0.0345 (7)	−0.0049 (9)	−0.0011 (8)	−0.0058 (6)
C1	0.0425 (7)	0.0344 (6)	0.0345 (6)	0.0132 (5)	−0.0005 (5)	−0.0023 (5)
C2	0.0411 (7)	0.0453 (7)	0.0339 (6)	0.0142 (6)	0.0007 (5)	−0.0009 (5)
C3	0.0522 (9)	0.0610 (9)	0.0448 (7)	0.0249 (7)	0.0025 (6)	−0.0059 (6)
C4	0.0465 (9)	0.0950 (14)	0.0521 (9)	0.0301 (9)	0.0098 (7)	0.0011 (9)
C5	0.0393 (8)	0.0861 (12)	0.0565 (9)	0.0080 (8)	0.0061 (7)	0.0083 (8)
C6	0.0434 (8)	0.0558 (8)	0.0473 (8)	0.0050 (6)	−0.0004 (6)	0.0041 (6)
C7	0.0390 (7)	0.0450 (7)	0.0345 (6)	0.0117 (5)	0.0005 (5)	0.0011 (5)
C8	0.0375 (7)	0.0338 (6)	0.0368 (6)	0.0085 (5)	0.0003 (5)	−0.0019 (5)
C23	0.0365 (6)	0.0374 (6)	0.0351 (6)	0.0113 (5)	0.0026 (5)	−0.0010 (5)
C20	0.0383 (7)	0.0374 (6)	0.0344 (6)	0.0100 (5)	0.0016 (5)	−0.0015 (5)
C21	0.0447 (7)	0.0383 (6)	0.0401 (7)	0.0097 (6)	−0.0012 (5)	0.0004 (5)
C22	0.0725 (12)	0.0807 (12)	0.0532 (9)	0.0335 (10)	0.0043 (8)	0.0249 (8)
C24	0.0514 (8)	0.0363 (6)	0.0380 (7)	0.0142 (6)	−0.0005 (6)	−0.0036 (5)
C25	0.0901 (15)	0.0779 (13)	0.0740 (12)	0.0069 (11)	−0.0338 (11)	−0.0254 (10)
C9	0.0450 (8)	0.0343 (6)	0.0446 (7)	0.0122 (6)	−0.0013 (6)	−0.0020 (5)
O2	0.1124 (11)	0.0517 (6)	0.0545 (7)	0.0423 (7)	−0.0019 (7)	−0.0020 (5)
O3	0.0697 (7)	0.0588 (6)	0.0388 (5)	0.0287 (5)	0.0024 (5)	0.0074 (4)
O4	0.0834 (9)	0.0810 (8)	0.0479 (6)	0.0093 (7)	0.0176 (6)	−0.0169 (6)
O5	0.0544 (7)	0.0663 (7)	0.0561 (6)	0.0035 (5)	−0.0083 (5)	−0.0156 (5)
O1	0.0372 (5)	0.0334 (4)	0.0382 (5)	0.0104 (4)	−0.0025 (4)	−0.0017 (3)
S1	0.0928 (13)	0.0521 (9)	0.0734 (13)	0.0312 (9)	0.0184 (10)	0.0206 (9)
C12	0.100 (8)	0.061 (5)	0.118 (8)	0.014 (4)	−0.009 (5)	0.016 (4)
C11	0.068 (4)	0.059 (6)	0.076 (6)	0.031 (5)	−0.001 (4)	−0.012 (4)
C10	0.087 (4)	0.035 (2)	0.072 (5)	0.027 (2)	−0.012 (4)	−0.001 (3)
S1'	0.0557 (7)	0.0439 (8)	0.0641 (7)	0.0204 (6)	0.0050 (6)	−0.0040 (6)
C12'	0.088 (6)	0.059 (6)	0.064 (6)	0.019 (4)	−0.011 (4)	−0.027 (4)
C11'	0.129 (7)	0.050 (5)	0.071 (5)	0.025 (4)	0.018 (4)	0.013 (4)
C10'	0.078 (5)	0.044 (4)	0.075 (6)	0.035 (4)	−0.005 (4)	−0.002 (3)

### Geometric parameters (Å, º)

**Table:**

C17—C16	1.511 (2)	C23—C20	1.3335 (17)
C17—H17A	0.9600	C23—C24	1.4831 (17)
C17—H17B	0.9600	C20—C21	1.4782 (18)
C17—H17C	0.9600	C21—O2	1.1969 (17)
C16—C15	1.377 (3)	C21—O3	1.3204 (17)
C16—C18	1.379 (2)	C22—O3	1.4465 (18)
C18—C19	1.377 (2)	C22—H22A	0.9600
C18—H18	0.9300	C22—H22B	0.9600
C19—C13	1.384 (2)	C22—H22C	0.9600
C19—H19	0.9300	C24—O4	1.1932 (18)
C13—C14	1.3881 (19)	C24—O5	1.3195 (19)
C13—C1	1.4970 (17)	C25—O5	1.453 (2)
C14—C15	1.380 (2)	C25—H25A	0.9600
C14—H14	0.9300	C25—H25B	0.9600
C15—H15	0.9300	C25—H21C	0.9600
C1—O1	1.4606 (14)	C9—C12	1.417 (9)
C1—C2	1.5353 (19)	C9—C12'	1.428 (10)
C1—C20	1.5515 (17)	C9—S1'	1.673 (3)
C2—C3	1.381 (2)	C9—S1	1.676 (3)
C2—C7	1.3984 (19)	S1—C10	1.697 (11)
C3—C4	1.395 (3)	C12—C11	1.392 (14)
C3—H3	0.9300	C12—H12	0.9300
C4—C5	1.374 (3)	C11—C10	1.338 (7)
C4—H4	0.9300	C11—H11	0.9300
C5—C6	1.399 (2)	C10—H10	0.9300
C5—H5	0.9300	S1'—C10'	1.696 (12)
C6—C7	1.374 (2)	C12'—C11'	1.390 (14)
C6—H6	0.9300	C12'—H12'	0.9300
C7—C8	1.5419 (19)	C11'—C10'	1.337 (7)
C8—O1	1.4498 (15)	C11'—H11'	0.9300
C8—C9	1.4853 (18)	C10'—H10'	0.9300
C8—C23	1.5521 (17)		
			
C16—C17—H17A	109.5	C24—C23—C8	124.05 (11)
C16—C17—H17B	109.5	C23—C20—C21	128.37 (11)
H17A—C17—H17B	109.5	C23—C20—C1	106.33 (11)
C16—C17—H17C	109.5	C21—C20—C1	122.53 (10)
H17A—C17—H17C	109.5	O2—C21—O3	125.26 (13)
H17B—C17—H17C	109.5	O2—C21—C20	122.09 (13)
C15—C16—C18	117.71 (14)	O3—C21—C20	112.60 (11)
C15—C16—C17	121.50 (17)	O3—C22—H22A	109.5
C18—C16—C17	120.79 (18)	O3—C22—H22B	109.5
C19—C18—C16	121.53 (16)	H22A—C22—H22B	109.5
C19—C18—H18	119.2	O3—C22—H22C	109.5
C16—C18—H18	119.2	H22A—C22—H22C	109.5
C18—C19—C13	120.66 (14)	H22B—C22—H22C	109.5
C18—C19—H19	119.7	O4—C24—O5	125.08 (13)
C13—C19—H19	119.7	O4—C24—C23	124.66 (14)
C19—C13—C14	118.14 (13)	O5—C24—C23	110.22 (12)
C19—C13—C1	119.86 (12)	O5—C25—H25A	109.5
C14—C13—C1	121.69 (13)	O5—C25—H25B	109.5
C15—C14—C13	120.44 (15)	H25A—C25—H25B	109.5
C15—C14—H14	119.8	O5—C25—H21C	109.5
C13—C14—H14	119.8	H25A—C25—H21C	109.5
C16—C15—C14	121.52 (15)	H25B—C25—H21C	109.5
C16—C15—H15	119.2	C12—C9—C12'	106.5 (9)
C14—C15—H15	119.2	C12—C9—C8	126.6 (5)
O1—C1—C13	109.03 (10)	C12'—C9—C8	126.9 (6)
O1—C1—C2	99.71 (10)	C12—C9—S1'	3.5 (6)
C13—C1—C2	122.54 (11)	C12'—C9—S1'	109.8 (6)
O1—C1—C20	98.77 (9)	C8—C9—S1'	123.20 (15)
C13—C1—C20	118.47 (11)	C12—C9—S1	110.4 (5)
C2—C1—C20	104.23 (10)	C12'—C9—S1	6.3 (6)
C3—C2—C7	120.66 (14)	C8—C9—S1	122.68 (15)
C3—C2—C1	134.05 (13)	S1'—C9—S1	113.69 (18)
C7—C2—C1	105.03 (11)	C21—O3—C22	116.20 (12)
C2—C3—C4	117.93 (15)	C24—O5—C25	115.45 (14)
C2—C3—H3	121.0	C8—O1—C1	97.66 (9)
C4—C3—H3	121.0	C9—S1—C10	92.5 (5)
C5—C4—C3	121.26 (15)	C11—C12—C9	111.6 (11)
C5—C4—H4	119.4	C11—C12—H12	124.2
C3—C4—H4	119.4	C9—C12—H12	124.2
C4—C5—C6	120.97 (16)	C10—C11—C12	112.9 (12)
C4—C5—H5	119.5	C10—C11—H11	123.6
C6—C5—H5	119.5	C12—C11—H11	123.6
C7—C6—C5	117.80 (15)	C11—C10—S1	112.4 (11)
C7—C6—H6	121.1	C11—C10—H10	123.8
C5—C6—H6	121.1	S1—C10—H10	123.8
C6—C7—C2	121.35 (13)	C9—S1'—C10'	93.2 (6)
C6—C7—C8	133.63 (13)	C11'—C12'—C9	111.2 (12)
C2—C7—C8	104.81 (11)	C11'—C12'—H12'	124.4
O1—C8—C9	111.21 (10)	C9—C12'—H12'	124.4
O1—C8—C7	100.06 (9)	C10'—C11'—C12'	113.4 (14)
C9—C8—C7	120.07 (11)	C10'—C11'—H11'	123.3
O1—C8—C23	98.86 (9)	C12'—C11'—H11'	123.3
C9—C8—C23	118.64 (11)	C11'—C10'—S1'	111.8 (13)
C7—C8—C23	104.50 (10)	C11'—C10'—H10'	124.1
C20—C23—C24	129.51 (12)	S1'—C10'—H10'	124.1
C20—C23—C8	105.73 (10)		
			
C15—C16—C18—C19	0.4 (3)	C2—C1—C20—C21	−92.07 (14)
C17—C16—C18—C19	180.0 (2)	C23—C20—C21—O2	−145.48 (17)
C16—C18—C19—C13	0.1 (3)	C1—C20—C21—O2	13.0 (2)
C18—C19—C13—C14	−0.5 (2)	C23—C20—C21—O3	32.2 (2)
C18—C19—C13—C1	173.23 (14)	C1—C20—C21—O3	−169.33 (12)
C19—C13—C14—C15	0.5 (2)	C20—C23—C24—O4	−73.8 (2)
C1—C13—C14—C15	−173.11 (15)	C8—C23—C24—O4	117.26 (17)
C18—C16—C15—C14	−0.4 (3)	C20—C23—C24—O5	108.36 (17)
C17—C16—C15—C14	−180.0 (2)	C8—C23—C24—O5	−60.61 (16)
C13—C14—C15—C16	−0.1 (3)	O1—C8—C9—C12	84.9 (7)
C19—C13—C1—O1	−81.69 (15)	C7—C8—C9—C12	−158.9 (7)
C14—C13—C1—O1	91.80 (15)	C23—C8—C9—C12	−28.6 (7)
C19—C13—C1—C2	162.74 (13)	O1—C8—C9—C12'	−93.7 (7)
C14—C13—C1—C2	−23.77 (19)	C7—C8—C9—C12'	22.5 (7)
C19—C13—C1—C20	30.06 (18)	C23—C8—C9—C12'	152.7 (7)
C14—C13—C1—C20	−156.45 (13)	O1—C8—C9—S1'	83.85 (18)
O1—C1—C2—C3	−152.34 (15)	C7—C8—C9—S1'	−159.97 (15)
C13—C1—C2—C3	−32.2 (2)	C23—C8—C9—S1'	−29.7 (2)
C20—C1—C2—C3	105.94 (16)	O1—C8—C9—S1	−88.1 (2)
O1—C1—C2—C7	33.71 (12)	C7—C8—C9—S1	28.1 (2)
C13—C1—C2—C7	153.82 (11)	C23—C8—C9—S1	158.34 (19)
C20—C1—C2—C7	−68.00 (12)	O2—C21—O3—C22	0.3 (2)
C7—C2—C3—C4	−1.3 (2)	C20—C21—O3—C22	−177.29 (13)
C1—C2—C3—C4	−174.46 (14)	O4—C24—O5—C25	5.3 (2)
C2—C3—C4—C5	1.6 (2)	C23—C24—O5—C25	−176.88 (14)
C3—C4—C5—C6	−0.3 (3)	C9—C8—O1—C1	−178.92 (11)
C4—C5—C6—C7	−1.4 (2)	C7—C8—O1—C1	53.14 (10)
C5—C6—C7—C2	1.8 (2)	C23—C8—O1—C1	−53.42 (10)
C5—C6—C7—C8	175.54 (14)	C13—C1—O1—C8	176.84 (10)
C3—C2—C7—C6	−0.4 (2)	C2—C1—O1—C8	−53.64 (10)
C1—C2—C7—C6	174.50 (12)	C20—C1—O1—C8	52.55 (11)
C3—C2—C7—C8	−175.79 (12)	C12—C9—S1—C10	2.8 (7)
C1—C2—C7—C8	−0.84 (13)	C12'—C9—S1—C10	−49 (7)
C6—C7—C8—O1	152.90 (14)	C8—C9—S1—C10	176.8 (4)
C2—C7—C8—O1	−32.60 (12)	S1'—C9—S1—C10	4.2 (5)
C6—C7—C8—C9	31.1 (2)	C12'—C9—C12—C11	3.9 (16)
C2—C7—C8—C9	−154.42 (11)	C8—C9—C12—C11	−174.9 (13)
C6—C7—C8—C23	−105.12 (16)	S1'—C9—C12—C11	−160 (11)
C2—C7—C8—C23	69.38 (12)	S1—C9—C12—C11	−1.2 (16)
O1—C8—C23—C20	34.38 (12)	C9—C12—C11—C10	−2 (2)
C9—C8—C23—C20	154.53 (12)	C12—C11—C10—S1	4 (2)
C7—C8—C23—C20	−68.50 (13)	C9—S1—C10—C11	−3.8 (15)
O1—C8—C23—C24	−154.44 (12)	C12—C9—S1'—C10'	20 (10)
C9—C8—C23—C24	−34.28 (18)	C12'—C9—S1'—C10'	3.0 (13)
C7—C8—C23—C24	102.68 (14)	C8—C9—S1'—C10'	−174.9 (11)
C24—C23—C20—C21	−10.6 (2)	S1—C9—S1'—C10'	−2.3 (12)
C8—C23—C20—C21	159.93 (13)	C12—C9—C12'—C11'	−7.9 (14)
C24—C23—C20—C1	−171.81 (13)	C8—C9—C12'—C11'	170.9 (9)
C8—C23—C20—C1	−1.28 (13)	S1'—C9—C12'—C11'	−6.9 (13)
O1—C1—C20—C23	−31.92 (13)	S1—C9—C12'—C11'	122 (7)
C13—C1—C20—C23	−149.24 (12)	C9—C12'—C11'—C10'	8 (2)
C2—C1—C20—C23	70.50 (13)	C12'—C11'—C10'—S1'	−6 (3)
O1—C1—C20—C21	165.50 (12)	C9—S1'—C10'—C11'	2 (2)
C13—C1—C20—C21	48.18 (18)		

### Hydrogen-bond geometry (Å, º)

Cg is the centroid of the C2–C7 ring.

**Table:**

*D*—H···*A*	*D*—H	H···*A*	*D*···*A*	*D*—H···*A*
C11—H11···O4^i^	0.93	2.47	3.378 (1)	165
C17—H17*B*···*Cg*^ii^	0.96	3.26	3.99 (2)	136

Symmetry codes: (i) −*x*, −*y*+1, −*z*+1; (ii) *x*+1, *y*, *z*.

## References

[bb1] Balakrishnan, B., Nandakumar, M., Seshadri, P. R. & Mohanakrishnan, A. K. (2013). *Acta Cryst.* E**69**, o323. 10.1107/S1600536813002791 PMC3588525 23476521

[bb2] Bruker (2008). *APEX2* and *SAINT* Bruker AXS Inc., Madison. Wisconsin, USA.

[bb3] Cremer, D. & Pople, J. A. (1975). *J. Am. Chem. Soc.* **97**, 1354–1358.

[bb4] Denmark, S. E. & Thorarensen, A. (1996). *Chem. Rev.* **96**, 137–166. 10.1021/cr940277f 11848747

[bb5] Farrugia, L. J. (2012). *J. Appl. Cryst.* **45**, 849–854.

[bb6] Fun, H.-K., Suwunwong, T. & Chantrapromma, S. (2011). *Acta Cryst.* E**67**, o701–o702. 10.1107/S1600536811006106 PMC3052174 21522446

[bb7] Gurbanov, A. V., Nikitina, E. V., Sorokina, E. A., Zubkov, F. I. & Khrustalev, V. N. (2009). *Acta Cryst.* E**65**, o3243–o3244. 10.1107/S1600536809050600 PMC2972130 21578945

[bb8] Nardelli, M. (1983). *Acta Cryst.* C**39**, 1141–1142.

[bb9] Ohwada, T., Miura, M., Tanaka, H., Sakamoto, S., Yamaguchi, K., Ikeda, H. & Inagaki, S. (2001). *J. Am. Chem. Soc.* **123**, 10164–10172. 10.1021/ja010917d 11603965

[bb10] Sheldrick, G. M. (2008). *Acta Cryst.* A**64**, 112–122. 10.1107/S0108767307043930 18156677

[bb11] Spek, A. L. (2009). *Acta Cryst.* D**65**, 148–155. 10.1107/S090744490804362X PMC2631630 19171970

[bb12] Takahashi, I., Tsuzuki, M., Kitajima, H., Hetanaka, M., Maeda, S., Yamano, A., Ohta, T. & Hosoi, S. (2003). *Anal. Sci.* **19**, 973–974. 10.2116/analsci.19.973 12834250

[bb13] Westrip, S. P. (2010). *J. Appl. Cryst.* **43**, 920–925.

